# Southern African guidelines on the safe, easy and effective use of pre-exposure prophylaxis: 2020

**DOI:** 10.4102/sajhivmed.v21i1.1152

**Published:** 2020-12-10

**Authors:** Linda-Gail Bekker, Benjamin Brown, Dvora Joseph-Davey, Kathrine Gill, Michelle Moorhouse, Sinead Delany-Moretlwe, Landon Myer, Catherine Orrell, Kevin Rebe, W.D. Francois Venter, Carole L. Wallis

**Affiliations:** 1 Desmond Tutu HIV Centre, Institute of Infectious Disease and Molecular Medicine, University of Cape Town, Cape Town, South Africa; 2 Anova Health Institute, Johannesburg, South Africa; 3 Department of Epidemiology, University of California, Los Angeles, United States of America; 4 Division of Epidemiology and Biostatistics, School of Public Health and Family Medicine, University of Cape Town, Cape Town, South Africa; 5 Wits Reproductive Health and HIV Research Unit, University of the Witwatersrand, Johannesburg, South Africa; 6 Department of Medicine, University of Cape Town, Cape Town, South Africa; 7 Life Vincent Pallotti Hospital, Cape Town, South Africa; 8 Department of Medicine and Infectious Diseases, University of Cape Town, Cape Town, South Africa; 9 Ezintsha, Faculty of Health Sciences, University of the Witwatersrand, Johannesburg, South Africa; 10 BARC-SA, Speciality Molecular Division, Lancet Laboratories, Johannesburg, South Africa

## Introduction

Pre-exposure prophylaxis (PrEP) with antiretroviral agents to prevent human immunodeficiency virus (HIV) acquisition is now a standard of care in many countries. After more than a decade of research and dozens of randomised trials, it is clear that PrEP is both safe and efficacious. Oral PrEP is thus a key component of an HIV prevention package and should be offered to anyone who may be exposed to HIV, whether sexually or through other means. With the highest HIV incidence in the world, PrEP use in the South African population remains unacceptably low and insufficient to reach its full impact as an HIV control measure. To realise the full value of this prevention tool, PrEP must become more accessible. Therefore, the updated 2020 PrEP guidelines have (1) broadened eligible groups to include pregnant and breastfeeding women, (2) reduced clinical and health system barriers to simplify PrEP initiation and administration (e.g. same-day PrEP), (3) broadened PrEP delivery to include on-demand PrEP in men who have sex with men and transgender women, (4) provided updates of adverse events and relevant drug–drug interactions and (5) suggested parameters with which to measure PrEP rollout and success.

## Background

The first Southern African HIV Clinicians Society pre-exposure prophylaxis (PrEP) guidelines were published in the Southern African Journal of HIV Medicine in 2012 following labelling approval by the Federal Drug Administration (FDA) in the United States of America.^1^ The results of three clinical trials underpinned those guidelines: the Global iPrEx study in men who have sex with men (MSM) and transgender (TG) people, the Partners PrEP study in discordant couples in Uganda and Kenya and the tenofovir disoproxil fumarate (TDF) 2 study in heterosexual men and women from Botswana.^2,3,4,5^ Since then a further seven randomised controlled trials (RCTs) and numerous open label demonstration studies have led to the registration of combination therapy, with tenofovir and emtricitabine or related variations thereof as effective tools in the prevention of human immunodeficiency virus (HIV) transmission to uninfected persons.^6,7,8,9,10,11,12,13,14,15,16,17,18,19^ The World Health Organization (WHO) set a target of 3 million PrEP users worldwide by 2020. With 240 000 incident HIV infections per year in South Africa, which is equivalent to almost 15% of all new infections globally,^20^ a significant portion of those effective PrEP users should be in this country. However, despite the research, demonstration projects and existing guidelines, PrEP use remains low and insufficient to effectively reduce South African HIV incidence rates, with only an estimated 45 000 people using PrEP as of June 2020.^21^

People who have been offered PrEP and have integrated it into their daily lives describe how they have felt more in control of their circumstances, more free of worry and able to once again enjoy their sexual intimacies in ways that have not been possible for decades given South Africa’s very high HIV prevalence. In foreign cities such as London and San Francisco and regions such as New South Wales in Australia where universal test and treat strategies have been coupled with extensive scale-up for PrEP, the rates of new HIV infections have dropped precipitously.^22^ It is expected that with the assistance of these guidelines, further PrEP scale-up will soon be possible in South Africa with similar positive outcomes.

**FIGURE 1:**
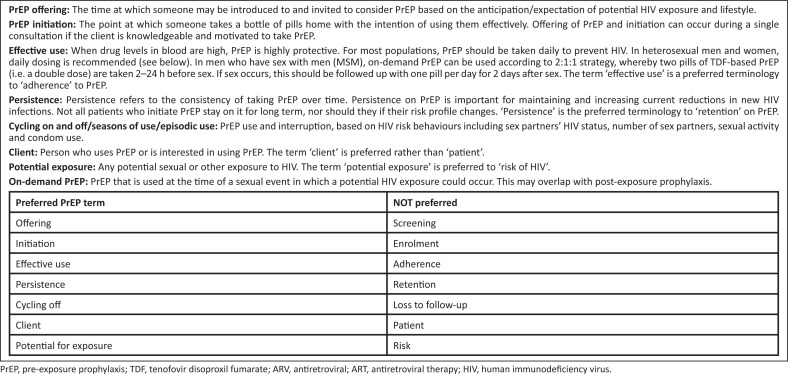
Pre-exposure prophylaxis introduces a new lexicon.

The 2012 PrEP guideline was last updated in 2016. The current (2020) guideline provides further options regarding drug use and the practice of oral PrEP,^1^ including (1) broadened eligible groups to include pregnant and breastfeeding women, (2) reduced clinical and health system barriers to simplify PrEP initiation and administration (e.g. same-day PrEP), (3) broadened PrEP delivery to include on-demand PrEP in MSM and TG women, (4) provided updates of adverse events and relevant drug–drug interactions and (5) suggested parameters with which to measure PrEP rollout and success. We also introduce alternative oral antiretroviral (ARV) agents and new modalities on the horizon. We present an updated lexicon for PrEP clients and users in Figure 1.

## Quick facts on oral pre-exposure prophylaxis

At this time, PrEP is the *daily* use by the HIV-uninfected of oral TDF or tenofovir alafenamide (TAF)/Emtricitabine (FTC) co-formulated with emtricitabine (TDF/FTC or F/TAF) or variations of this, for example, TDF on its own or co-administered with lamivudine (3TC) to prevent HIV acquisition (transmission). The most commonly used preparation and the one licensed in South Africa for oral PrEP is TDF/FTC. Pre-exposure prophylaxis has been shown to be effective amongst a wide range of HIV-negative populations. There are other drugs and other routes of administration under investigation, for example, a topical dapivirine vaginal ring and long-acting injectable cabotegravir. The registration of TAF is currently under review in South Africa. These guidelines will be updated as new data become available^21^:

- Pre-exposure prophylaxis has a long history of effectiveness in the setting of preventing vertical HIV transmission. Protective *in utero* foetal ARV drug levels are optimised prior to delivery (exposure).^23^
- Consistent adherence to PrEP reduces the risk of HIV transmission from sex by > 95%.^2^
- For those at risk, daily PrEP has been confirmed to be effective in the prevention of sexual and injecting drug use HIV transmission. Where adherence is suboptimal, PrEP is less effective (unreliable) as protective drug (ARV) levels at the time and site of exposure may be too low. Daily use is the most dependable way to ensure effectiveness.^24^
- Condom use is still recommended as PrEP does not protect against other sexually transmitted infections (STIs), such as syphilis, chlamydia and gonorrhoea.
- Pre-exposure prophylaxis has no contraceptive effect. The drugs used in PrEP do NOT interact with hormonal contraception.
- Pre-exposure prophylaxis is safe to take when pregnant or breastfeeding.^19,25^
- **On-demand PrEP (for MSM and TG women only):** Two pills are taken 2–24 h before sex. If sex occurs, the individual who is or is presumed to be HIV-uninfected follows up with one pill per day for 2 days after sex.^26^
- Pre-exposure prophylaxis is generally well tolerated. Occasional side effects include nausea, bloating and/or headaches in approximately one in 10 users.
- Whilst TDF/FTC is available for all populations, TAF/FTC has so far only been shown to be efficacious in men and TG women. Studies in cisgender women are planned.
- Pre-exposure prophylaxis at this time is in the form of oral pills only, but topical rings and long-acting injectables are under investigation.^27^

### Who is pre-exposure prophylaxis for?

Pre-exposure prophylaxis is an effective prevention option for any sexually active person who might be exposed to HIV through contact with HIV-infected body fluids (genital and blood). Pre-exposure prophylaxis is suitable for people of any sex, gender and sexual orientation.

The WHO recommends that PrEP should be scaled up for populations where the incidence of HIV is 3% or greater.^24^ Although risk is unevenly distributed across sub-populations and geographic areas in Southern Africa, a very large number of sexually active people are exposed to this degree of risk. Whilst many PrEP efforts have focused on specific ‘high-risk’ or key population groups, at an individual client level, anyone who reports that he or she is at risk of HIV infection might benefit from PrEP. In these cases, PrEP education should be provided and intervention should be offered.

On the contrary, the use of a risk scoring tool is not recommended but rather that an accurate sexual history is elicited from clients to identify sexual behaviours that justify consideration of improved or enhanced HIV prevention strategies. This is because risk scores fail when risk is not well judged and individual risk levels are dynamic; in addition, no single risk score has been validated for generalised use. Given the high ongoing rates of HIV transmission in South Africa and low current PrEP demand and no saturation in both the private and public sectors,^21^ people seeking PrEP should be encouraged to initiate PrEP, provided that they are sexually active and there is a reasonable risk that they might be exposed to HIV (see Table 1).

**TABLE 1:** Populations for pre-exposure prophylaxis consideration.

Population	Risk group	Special considerations
Adolescents	Any/all	**Must weigh > 35 kg**. Should be allowed to consent independently but support is advisable especially to assist with persistence.
Women	Mostly vaginal sex but may also engage in oral† and anal sex	Pre-exposure prophylaxis (PrEP) is highly efficacious when dosed daily in HIV-uninfected women. It is a user-dependent, discreet addition to the prevention menu for women. When providing PrEP to women, it is important to provide it in the context of other health interventions.^27,28,29^ Considerations such as pap smears, contraception, breastfeeding and post-partum care should be covered. However, access to these is not a prerequisite to prescribing PrEP.
Men	Penile and oral	Pre-exposure prophylaxis works for men who are HIV-negative and at risk of HIV acquisition.
Men who have sex with men (MSM)	Penile, anal and oral	Tissue concentrations of TDF/FTC appear to be higher at the anal mucosa and are reached more rapidly in the anal mucosa than in vaginal mucosa. It has been demonstrated in modelling studies that four doses per week may be sufficient to safely protect MSM. Recent trials have also confirmed that on-demand PrEP is efficacious.^28^
Pregnant and breastfeeding women	Primarily vaginal sex but may also engage in anal and oral sex	Pre-exposure prophylaxis is safe in pregnancy and during lactation. There are no contraindications of taking PrEP during pregnancy and breastfeeding.^19,25^ HIV incidence is high during pregnancy and breastfeeding, with HIV acquisition risk more than double during pregnancy and the postpartum period compared to when women are not pregnant.^30^ Pre-exposure prophylaxis counselling should be provided to all HIV-negative pregnant women at risk of HIV. Pre-exposure prophylaxis provision and risk reduction counselling should be aligned with antenatal and postnatal visits. Symptoms such as nausea and gastrointestinal symptoms are far more common and more severe than with PrEP, especially in the first trimester, and should thus be actively managed. There are no data yet on TAF/FTC in pregnancy.
Serodiscordant couples	A partner has unknown or HIV-positive status and is not virally suppressed	Pre-exposure prophylaxis may be used as a ‘bridge’ until the partner living with HIV has an undetectable viral load – at that point PrEP may be discontinued depending on the preference of the couple.^31^
Safer conception	Serodiscordant couples wishing to conceive	Pre-exposure prophylaxis may be provided to the HIV-negative partner during condomless sex whilst trying to conceive, and whilst pregnant and breastfeeding.^32,33,34,35^ Pre-exposure prophylaxis should be continued until the partner living with HIV has initiated ART and achieved viral suppression (viral load < 200 copies/mL).
Drug using individuals	Needle sharing caries high HIV risk	Pre-exposure prophylaxis has been shown to be effective in one large RCT and some demonstration studies of intravenous drug using populations of both sexes.
Transgender people	Anal and oral sex	Transgender (TG) women have very high rates of HIV acquisition and PrEP is effective although specific evidence is limited.

MSM, men who have sex with men; PrEP, pre-exposure prophylaxis; TDF, tenofovir disoproxil fumarate; RCT, randomised controlled trial; TAF, tenofovir alafenamide; FTC, emtricitabine; ART, antiretroviral therapy; HIV, human immunodeficiency virus.

† , Oral sex involves using the mouth to stimulate the penis (fellatio), vagina (cunnilingus) or anus (anilingus). The chance an HIV-negative person will get HIV from oral sex with an HIV-positive partner is extremely low and lower than anal or vaginal sex. The risk of HIV transmission through oral sex is even lower if the HIV-negative partner is taking PrEP.

Differing pharmacokinetic (PK) data play a role in different recommendations for dose frequency in different populations. Tissue drug concentrations in genital and anal mucosa vary with higher levels and steady states reached more rapidly in anal compared with vaginal mucosa.^28^ Pharmacokinetic modelling studies have suggested that fewer doses may be required to reach effective concentrations in anal compared with vaginal mucosa. This has led to three recommendations that depend on whether exposure is via vaginal (heterosexual sex) or anal mucosal routes:

- Pre-exposure prophylaxis where the HIV exposure is via vaginal mucosa should be dosed daily.
- Pre-exposure prophylaxis where the HIV exposure is via vaginal mucosa may require up to 7 days of dosing *before* being fully effective.
- On-demand PrEP is not recommended where exposure is via vaginal mucosa.

### When should pre-exposure prophylaxis not be offered?

- Pre-exposure prophylaxis should not be offered to anyone who is suspected or confirmed to be HIV-positive. Providing PrEP to an individual who is HIV-positive or acutely seroconverting is sub-optimal treatment for HIV and could lead to antiviral drug resistance.
- Individuals who refuse to HIV test should be counselled and PrEP should not be offered.
- Pre-exposure prophylaxis should be delayed in anyone with an acute viral illness that could be because of HIV seroconversion. There is considerable overlap in symptoms and signs caused by viruses; therefore, any potential PrEP client presenting with fever, myalgia, arthralgia, rash, headache, and oral or genital ulcers might be HIV-positive but in the window period. Other HIV prevention options, like condoms, should be discussed, repeat testing should be arranged for 2 weeks later, with PrEP offered then if repeat test is negative.
- Tenofovir-based PrEP should not be offered to anyone with pre-existing renal dysfunction (Estimated glomerular filtration rate *i* [eGFR] < 50 mL/min). Clients can return in 1–3 weeks to re-test eGFR to re-assess eligibility.
- Individuals < 35 kilograms (kg) should not be given oral PrEP.

### Simplifying pre-exposure prophylaxis to improve access and optimise use

#### Pre-exposure prophylaxis is safe, well tolerated and easy to administer

**Step 1: Check client desirability of pre-exposure prophylaxis:** The aims of initiation consultations for PrEP are:

- **Understanding and insight of potential PrEP user:** To ensure that the PrEP user understands what PrEP is and the protection it provides, and has a personal plan for its effective use
- **Human immunodeficiency virus-negative status of user:** To ensure that the PrEP user is confirmed to be HIV-negative (rapid HIV testing acceptable)
- **Suitability and safety of PrEP for user:** To assess the suitability and safety of PrEP in those with renal and/or other potential contraindications.

**Step 2: Test for human immunodeficiency virus status:** Human immunodeficiency virus testing is required at initiation and at least 3 monthly whilst on pre-exposure prophylaxis to confirm HIV-negative status

- Follow HIV testing guidelines.
- Elicit a medical history and conduct a targeted examination to exclude acute exposure (symptoms suspicious of acute infection may be followed with repeat testing after 2 weeks to confirm HIV-negative status).
- Human immunodeficiency virus testing is advised 3–6 monthly whilst on PrEP to ensure breakthrough infection has not occurred.
- Human immunodeficiency virus self-testing may be used as an alternative whilst on PrEP.
- Inconclusive HIV test results should be referred for confirmatory testing.
- Pre-exposure prophylaxis should be stopped immediately in anyone with a positive or indeterminate HIV test result.
- Should an interruption in PrEP occur, then initiation testing should be performed (as above) prior to restart.

**Step 3: Check general well-being: Clinical assessment:** A clinical assessment for STIs should be performed at initiation, 6 monthly or when indicated.

- Appropriate STI screening is recommended and aetiologic testing and treatment should be provided when available. This should include nucleic acid antigen testing for *Chlamydia trachoma* and *Neisseria gonococcus* and serology for *Treponema pallidum*.
- Syndromic STI screening and management is otherwise recommended.
- Viral hepatitis B screening is recommended at PrEP initiation and screening if status is unknown.
- Hepatitis B vaccination is recommended if available or if screening serology test is negative.

**Step 4: Check for contraindications: Renal function:** A baseline assessment of renal function should be performed (creatinine and eGFR) in patients who are above 40 years of age, have co-morbidities or are on concomitant medication. Pre-exposure prophylaxis should not be used in people with a baseline eGFR of < 50 mL/min. Renal function may be checked annually and more frequently as dictated by an underlying renal problem or comorbidity.

**Step 5: Plan follow-up visits:**

- Assess how pill-taking is going for PrEP user.
- Interactions should be supportive and affirming.
- Identify a motivator to support effective pill-taking.
- Provide PrEP education regarding effective use and effectiveness of PrEP.
- Identify barriers to effective use.
- Provide realistic strategies to address barriers.
- Discuss use of other HIV prevention measures that are relevant to situation.
- Review need for PrEP and any change in sexual risk.

**Step 6: Package of prevention:** Providers can provide PrEP on the same day as counselling, following HIV testing. Pre-exposure prophylaxis alone provides high levels of HIV prevention; however, additional benefits are likely to accrue if it is offered as part of a *package of combination prevention* that includes:

- Counselling on effective use, starting and stopping PrEP.
- Agreement for follow-up HIV testing.
- Human immunodeficiency virus testing and counselling of sex partners (including HIV self-screening)
- Commodities such as condoms and sexual lubricants.
- Sexual health screening, including STI symptom check, aetiological STI testing if available and treatment either syndromically or as per laboratory results.
- Discussions on reproductive intent and provision of contraception as needed.
- Active safer conception counselling and guidance should be offered to women and couples who wish to conceive (see safer conception guidelines).
- Gender affirming counselling and treatment for TG populations.
- Immediate access to antiretroviral therapy (ART) for potential PrEP users who screen HIV-positive and require treatment.
- A prescription for PrEP (or PrEP medication) should be provided for a 3-month start.
- Adolescents and younger users or those who have identified pill-taking difficulties may be invited to return after 1 month to troubleshoot adherence difficulties.
- Telephonic contact may help with mild side effect management and difficulties with establishing pill-taking routines.
- A follow-up visit for clinical monitoring, counselling on persistence at 3 months, and then every 6 months or as required. Again, younger users may benefit from more regular contact.

#### Tips to support pre-exposure prophylaxis pill-taking

- Schedule medication taking time to correspond with the client’s daily routine activities (e.g. brushing teeth, eating breakfast and going to bed).
- Take pills at night if worried about side effects (e.g. in pregnant women).
- Use reminders, for example, cell phone, alarms, beepers and calendars.
- Use pillboxes to ensure daily use.
- Review disclosure issues to identify those who can support the client’s intentions to take their pills or barriers to pill-taking because of lack of disclosure or privacy at home.
- Join an online support group, for example, Facebook: PrEP Rethinking HIV Prevention.

#### Other considerations

Stopping and starting pre-exposure prophylaxis: Unlike taking ART, PrEP is not a lifelong intervention and individuals should be encouraged to ascertain risk and gauge their own need for PrEP. Different types of prevention may also be preferred at different times, for example, a holiday away versus busy working period at home.

Individuals should be instructed how to begin and stop daily use PrEP.

This is different from ‘on-demand’ PrEP, which is described in more detail below.

Tenofovir disoproxil fumarate/FTC can only prevent HIV if provided at sufficient levels in the tissues at the time of HIV exposure. The need for loading doses has been controversial and largely informed by PK modelling studies. The current research suggests that as many as 7 days of oral doses may be required in the case of vaginal mucosal exposure to ensure that sufficient tissue levels have been reached. However, clinical use in cis-males and trans-women suggests that high levels of protection can be achieved with dosing just before exposure.

If a PrEP user’s risk changes, that is, declines, or one wishes to stop PrEP for any reason, it should be affirmed that PrEP is not a lifelong intervention and that it is fine to stop. It is advised to take PrEP for up to 28 days after the last potential exposure to HIV (although this is not based on clinical evidence and alternative HIV prevention advice and commodities should be discussed). Clients should be invited to return to discuss PrEP at any point in future.

**Risk disinhibition:** Pre-exposure prophylaxis is highly efficacious and therefore it is unlikely even with more condomless sex that HIV infection will occur. Most studies have shown that increased access to care has resulted in less risky sex but in practice PrEP may result in more STIs and unintended pregnancies. For effective PrEP services, STI screening, appropriate treatment and prevention as well as contraception should be offered as part of an integrated sexual and reproductive health service at each PrEP clinical consultation.

**Sexually transmitted infections and pre-exposure prophylaxis:** Where feasible, a sexual history and a targeted examination is recommended to guide further screening and management, taking into account that STIs occur at all anatomic sites including oral, vaginal, penile and anal sites. The frequency of screening should be individualised and guided by the sexual history. We recommend that STI screening should occur at least annually and more frequently (6 monthly) in key populations such as MSM,^36,37^ pregnant women^38,39,40^ and sex workers. High rates of asymptomatic *Chlamydia trachomatis* (CT) are occurring amongst young women and MSM in the region. For this reason, where possible, nucleic acid amplification test (NAAT) testing for gonorrhoea and CT are highly recommended, but these tests are expensive and not always available. Syndromic STI screening and management should be offered as an alternative.

**Post-exposure prophylaxis to pre-exposure prophylaxis:** Individuals who frequently require post-exposure prophylaxis (PEP) for HIV exposure may benefit from PrEP. On completion of 28 days of triple ARV PEP therapy, oral PrEP may be continued with ongoing maintenance as before. Individuals who have a break between PEP and PrEP initiation should initiate as recommended above.

### Broaden pre-exposure prophylaxis modalities to include on-demand pre-exposure prophylaxis in men who have sex with men and transgender women

#### On demand pre-exposure prophylaxis

There is now robust evidence from the Intervention Préventive de l’Exposition aux Risques avec et pour les Gays (IPERGAY), and Intervention Préventive de l’Exposition aux Risques avec et pour les Gays (IPERGAY) Open Label Extension (OLE) and Prevenir studies^26,41^ that on-demand (i.e. sex or coital based dosing of PrEP) is effective for MSM and TG women and can be used as an alternative to daily dosing.

On-demand PrEP involves the so-called ‘2:1:1 strategy’. PrEP users are advised to take two pills of TDF-based PrEP (i.e. a double dose) 2–24 h before sex. If sex occurs, they should follow up with one pill per day for the following 2 days after sex.

This strategy allows minimisation of unnecessary PrEP doses when HIV exposure is unlikely (no sex) and therefore might decrease the risk of cumulative side effects. The strategy might suit individuals who do not want to take pills daily, allowing prevention doses to be focused around the time of HIV exposure risk. Should someone initiate on-demand PrEP, they should be counselled on the strategy and similar initiation precautions and investigations should be done. Human immunodeficiency virus status should be confirmed as negative, they should be considered for renal function testing and should attend their health provider regularly for STI screening and repeat HIV testing. New prescriptions should be administered as often as required.

#### Newer pre-exposure prophylaxis options

A recently added ARV shown to be effective for oral PrEP is a tenofovir (TFV) pro-drug called TAF that is approved in combination with other ARV agents for the treatment of HIV-1 infection in adults and paediatric patients. Tenofovir alafenamide has PK properties that distinguish it from TDF, resulting in clinically meaningful benefits that improve safety and increase the efficacy of TAF over TDF in PrEP. The lower levels of circulating TFV have consistently been associated with improved measures of renal and bone safety laboratory markers. Emtricitabine + TAF fixed dose combination pill (F/TAF) was shown in the recently published Emtricitabine and tenofovir alafenamide vs emtricitabine and tenofovir disoproxil fumarate for HIV pre-exposure prophylaxis (DISCOVER) trial to be non-inferior to F/TDF and has thus been licensed by the FDA for oral PrEP use in men and TG women.^42^ An equivalent trial is being designed for cisgender women in which TAF use in pregnancy will also be explored.

Long-acting cabotegravir which is a depot injectable integrase PrEP agent has just been shown to be non-inferior to oral TDF/FTC in a randomised clinical trial of MSM and TG women (HPTN 083).^43^ The companion study of cabotegravir long acting in African women is still underway (HPTN 084, the Life Study, NCT03164564).^i^

Finally, the topical dapivirine vaginal ring has just been recommended by the European Medical Agency as a PrEP intervention for women unable to safely utilise oral PrEP. This preventive tool was shown to reduce HIV acquisition by about 30% in women at risk of HIV acquisition in two RCTS conducted in Africa.^44^

### Updates to adverse events and drug–drug interactions

#### Adverse events

Tenofovir disoproxil fumarate and TAF are safe and well-tolerated drugs. Side effects do not occur in 90% or more clients who start PrEP. Initial minor side effects including headache and gastrointestinal upset (i.e. diarrhoea, nausea and loss of weight) may be experienced in up to 10% of people taking PrEP, but are self-limiting, with resolution within 2–3 weeks.^3^ These can be managed symptomatically. Tolerance improves over time.

**Renal toxicity:** A creatinine clearance (CrCl) test is recommended at the time of PrEP commencement to exclude asymptomatic renal disease but is not essential in well individuals under the age of 40 years and *should not delay PrEP start.* Tenofovir may cause a 5 mL/min – 6 mL/min reduction in CrCl in the first few months of use and if this prompts a PrEP pause, PrEP may be re-introduced in most cases without further problems.

In pregnant women, individuals > 40 years of age, those with a chronic disease and those using concomitant medications, creatinine should be drawn the same day as PrEP start (results can be communicated later) and repeated at months 6 and 12. More frequent monitoring of renal function may be required for people with chronic diseases such as hypertension and diabetes, as per the plan for that comorbidity. Tenofovir disoproxil fumarate should not be commenced if the CrCl is < 50 mL/min, and should be stopped if the CrCl declines below 50 mL/min. The client can re-test within 1 month to establish if their CrCl changes and can start PrEP then. Where renal toxicity is an issue, TAF/FTC may be considered as an alternative agent because of its renal sparing properties (see Table 2).

**TABLE 2:** Creatinine monitoring with tenofovir disoproxil fumarate pre-exposure prophylaxis.

Variable	At PrEP start	At PrEP follow-up
Well individual, ≤ 40 years	Recommended, not essential	Not required
> 40 years	Recommended	6 and 12 months
Pregnant	Recommended	6 and 12 months; not required after pregnancy if ≤ 40 years
Comorbidities	Recommended	6 and 12 months
Concomitant chronic medication	Recommended and essential or contra-indicated if nephrotoxic concomitant medication	6 and 12 months/contraindicated

PrEP, pre-exposure prophylaxis.

**Bone mineral density:** There is evidence for bone density loss with long-term use of TDF. For those with risk factors for reduced bone mineral density (BMD) (e.g. adolescents, people using recreational drugs such as amphetamines, people > 60 years of age, with known low BMD, post- and peri-menopausal women and those with a history of fragility fractures), the use of TAF or episodic TDF (to reduce exposure) could be considered.

**Hepatitis B:** Tenofovir disoproxil fumarate is also an antiviral treatment for hepatitis B. For this reason, screening for hepatitis B surface antigen is recommended prior to starting PrEP, but should not prevent PrEP start. Hepatitis B infection is also not a contraindication for PrEP use in individuals who would benefit. Caution when stopping PrEP may be required in those who are hepatitis B surface antigen positive. Rebound of hepatitis B virus resulting in liver injury has been described in the setting of ART and not PrEP but remains a theoretical concern. Hepatitis B vaccination is recommended for those who are hepatitis surface antigen negative.

**Drug resistance:** Drug resistance mostly occurs when PrEP is initiated at a time when the client is acutely HIV infected and is seroconverting. During these times, viral replication occurs rapidly in the blood. Pre-exposure prophylaxis drug concentrations are still suboptimal. Clients who seroconvert should stop PrEP use immediately and initiate ART as soon as possible. Monitoring of ART should follow adult treatment guidelines.

#### Drug–drug interactions

Transgender women on feminising hormonal treatment were thought to be in danger of drug–drug interactions with reduced efficacy of PrEP; however, a recent study has shown this is not the case.^45^

Tenofovir disoproxil fumarate is largely eliminated by the kidneys. There are few drug interactions of note, but TDF should be used with caution with medications that cause renal toxicity (see Table 3).

**TABLE 3:** Drug interactions with tenofovir disoproxil fumarate.

Drug name	Interaction	Response
Aminoglycosides (e.g. amikacin and gentamicin used in drug-resistant TB)	Possible additive nephrotoxicity	Avoid concomitant TDF

TDF, tenofovir disoproxil fumarate; TB, tuberculosis.

## Conclusion

We expect guidelines to be updated on a regular basis in line with ongoing research on vaginal rings, new drugs (including TAF), new regimens and injectable PrEP. South Africa is involved in several clinical trials. Longer term and, on-demand modalities are compelling alternatives for individuals who either do not want to take a daily pill and, or want to take PrEP intermittently. Emerging modalities such as vaginal films, microneedles and subdermal implants have numerous advantages but are still in early stages of development. Oral PrEP is a discreet, user-dependent, safe and effective prevention modality which is now part of the South African standard of HIV prevention. Adolescents and adults who deem themselves to be at risk of acquiring HIV can be offered this modality to enable safer sexual activity and worry-free intimate relationships. These guidelines will help simplify PrEP delivery to ensure that PrEP is available to all who need it.

## Footnotes

i With regard to long-acting single-agent injectable antiretroviral cabotegravir in the pre-exposure prevention (PrEP) of HIV infection/transmission to HIV-uninfected women (Study HPTN 084) and men (Study HPTN 083), both demonstrate superior efficacy versus standard oral PrEP. In the HPTN 084 Study, cabotegravir given every two months was 89% more effective than daily pills at preventing HIV acquisition. London, 9 November 2020. https://clinicaltrials.gov/ct2/show/NCT03164564. (HTPN 084); https://clinicaltrials.gov/ct2/show/NCT02720094. (HTPN 083). **Editor’s comment:** Note that this data will still require approval from international and local agencies. Cabotegravir is not currently registered for use in South Africa.
