# Corrigendum: Southern African guidelines on the safe, easy and effective use of pre-exposure prophylaxis: 2020

**DOI:** 10.4102/sajhivmed.v22i1.1295

**Published:** 2021-12-08

**Authors:** Linda-Gail Bekker, Benjamin Brown, Dvora Joseph-Davey, Kathrine Gill, Michelle Moorhouse, Sinead Delany-Moretlwe, Landon Myer, Catherine Orrell, Kevin Rebe, W.D. Francois Venter, Carole L. Wallis

**Affiliations:** 1 Desmond Tutu HIV Centre, Institute of Infectious Disease and Molecular Medicine, University of Cape Town, Cape Town, South Africa; 2 Anova Health Institute, Johannesburg, South Africa; 3 Department of Epidemiology, University of California, Los Angeles, United States of America; 4 Division of Epidemiology and Biostatistics, School of Public Health and Family Medicine, University of Cape Town, Cape Town, South Africa; 5 Wits Reproductive Health and HIV Research Unit, University of the Witwatersrand, Johannesburg, South Africa; 6 Department of Medicine, University of Cape Town, Cape Town, South Africa; 7 Life Vincent Pallotti Hospital, Cape Town, South Africa; 8 Department of Medicine and Infectious Diseases, University of Cape Town, Cape Town, South Africa; 9 Ezintsha, Faculty of Health Sciences, University of the Witwatersrand, Johannesburg, South Africa; 10 BARC-SA, Speciality Molecular Division, Lancet Laboratories, Johannesburg, South Africa

In the version of the article initially published, Bekker L-G, Brown B, Joseph-Davey D, et al. Southern African guidelines on the safe, easy and effective use of pre-exposure prophylaxis: 2020. S Afr J HIV Med. 2020;21(1), a1152. https://doi.org/10.4102/sajhivmed.v21i1.1152, the ORCID of the second last author was given incorrectly. The correct ORCID should be https://orcid.org/0000-0002-4157-732X instead of https://orcid.org/0000-0002-6919-5171 in the ‘Authors’ section.

This correction does not alter the study’s findings of significance or overall interpretation of the study’s results. The authors apologise for any inconvenience caused.

